# Ammonia distribution characteristics at the selective catalytic reduction reactor inlet with linear partitioning

**DOI:** 10.1016/j.isci.2024.111588

**Published:** 2024-12-12

**Authors:** Jiangdong Zhu, Zongquan Ye, Dehong Gong, Qian Wang, Qingling Luo

**Affiliations:** 1 Electrical Engineering College, Guizhou University, Guiyang 550025, China

**Keywords:** Catalysis, Engineering

## Abstract

Analyzing the uniformity of ammonia distribution at the inlet of selective catalytic reduction reactors is crucial for enhancing denitrification efficiency. To minimize ammonia slip while ensuring effective denitrification, this study examines ammonia flow characteristics in the SCR system under various zoning schemes. In scheme I, zones A1, A2, A3, and A4 predominantly influence the left, center, center-right, and far-right regions of the reactor inlet. As ammonia velocity increases from 4 m/s to 12 m/s, the concentration significantly increases, with Zone A4’s peak concentration rising from 0.0099 mol/m³ to 0.03 mol/m³. Similarly, increasing the ammonia spraying concentration from 1% to 9% enlarges the affected regions, particularly in Zone A1, where the impacted area expands from 1/3 to 1/2. Scheme II demonstrates a broader and more uniform distribution, which reduces localized concentrations but compromises precision in specific regions. This is of significant importance in reducing nitrogen oxide emissions in coal-fired power plants.

## Highlights

• Ammonia concentration distribution at the inlet of the SCR reactor
• Introduced a new concept of precise zonal ammonia injection
• Potential applications in reducing NOx emissions from coal-fired power plants

## Graphical abstract

**Figure:**
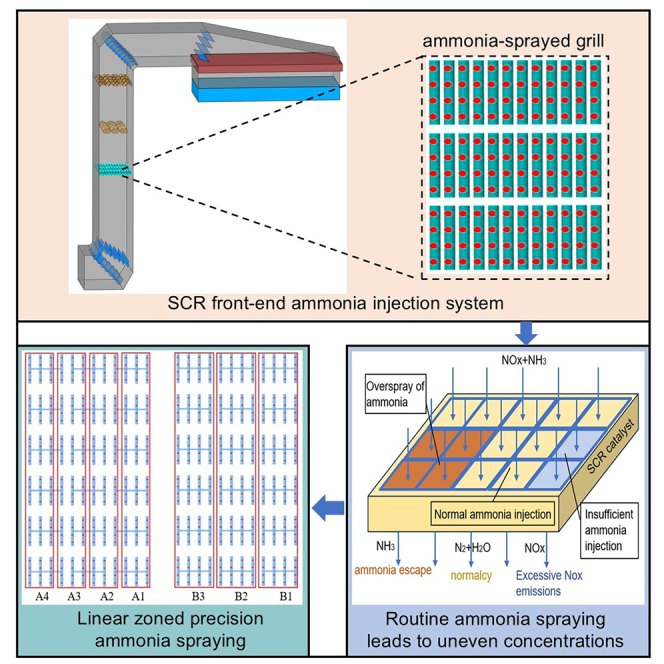

## Introduction

The rapid advancement of modern industry has led to a corresponding surge in energy consumption.^1^ In response to this challenge, researchers are exploring a range of clean energy technologies, including wind energy, solar photovoltaic cells,^2^ hydroelectric power, biomass energy, and high-efficiency energy storage solutions such as supercapacitors^3^ and batteries.^4^^,^^5^^,^^6^ In China’s strategic blueprint for sustainable economic development, renewable energy utilization occupies a pivotal role.^7^^,^^8^ However, due to the central role of coal-fired power in regulating the country’s energy supply, the fossil-fuel-dominated energy structure is expected to remain stable in the short term.^9^^,^^10^^,^^11^ Enhancing the clean and efficient utilization of coal power is therefore critical to the optimization and upgrading of China’s energy structure.^12^^,^^13^ The combustion of fossil fuels generates toxic gases, including nitrogen oxides (NOx) and sulfur dioxide (SO_2_), which contribute significantly to environmental pollution.^14^^,^^15^ Selective catalytic reduction (SCR) technology, which is the current mainstream method for flue gas denitrification, effectively controls pollutant emissions.^16^^,^^17^^,^^18^ In response to stringent environmental protection requirements, coal-fired power units in China have generally undergone ultra-low emission retrofits. However, following these retrofits, the SCR denitrification systems have demonstrated issues such as inaccurate ammonia injection control and uneven flow field distribution, resulting in localized ammonia slip and NOx emission exceedances. This has caused a dual negative impact on both the economic efficiency and environmental sustainability of the power units.^19^^,^^20^^,^^21^

In response to the challenges that emerged following the installation of SCR denitrification devices, numerous scholars have investigated the denitrification performance and internal flow characteristics of SCR reactors through experimental and numerical simulation methods.^22^^,^^23^^,^^24^ Given the challenges of obtaining data from real-world experiments, numerical simulation is favored as an alternative method due to its convenience and broad applicability.^25^^,^^26^^,^^27^^,^^28^ Research has demonstrated^29^ that the uniformity of the mixed gas flow field at the entrance of the SCR reactor significantly impacts system performance. The uniformity of the mixed flue gas flow field encompasses two key factors: the uniform distribution of gas velocity and the uniform distribution of the reducing agent NH₃. Of these, the uniform distribution of gas velocity exerts a greater influence on the catalyst’s denitrification performance. Furthermore, studying this aspect through numerical simulation is relatively convenient. As a result, current numerical simulation studies on SCR denitrification systems primarily focus on this area. Cong et al.^30^ simulated an SCR system for a 600 MW unit and significantly improved the velocity and ammonia distribution at the catalyst inlet cross-section by installing flow guiding plates in the flue expansion and deflection sections. Li et al.^31^ optimized the number and shape of flow-guiding plates in the gradual expansion section at the entrance of the SCR flue, effectively reducing the velocity distribution deviation across the catalyst section. Shang et al.^32^ analyzed the flow characteristics of flue gas within the SCR denitrification reactor and found that the complex internal structure of the SCR system led to flue gas recirculation. In the absence of flow-guiding plates, flow dead zones were observed within the catalyst layer. Wang et al.^33^ demonstrated that simulating different arrangements of guide vanes can improve the uniformity of flue gas velocity and enhance denitrification efficiency. Building on the optimization of the flow field, several studies have also analyzed the distribution of the ammonia-to-nitrogen ratio at the entrance of the SCR reactor. Yin et al.^34^ adjusted flue gas temperature and flow rate to regulate NH₃ concentration at the inlet, thereby reducing NOx emissions and NH₃ retention. Liu et al.^35^ used computational fluid dynamics to analyze the flux distribution of NOx in the cross-sectional area before NH₃ injection, contributing to improved SCR denitrification performance and reduced ammonia slip. Zeng et al.^36^ used numerical simulations to optimize the geometric structure of baffles, resulting in a more uniform distribution at the catalyst inlet post-optimization. Wang et al.^37^ and Wang and Li^38^ optimized the design of flow guiding plates and straightening plates, significantly improving the uniformity of the flue gas flow field and the ammonia-to-nitrogen ratio distribution.

The aforementioned studies have primarily focused on the uniformity of flue gas velocity distribution at the entrance of the SCR reactor, while neglecting the uniformity of ammonia distribution. However, the flow characteristics of ammonia are critical to velocity distribution at the reactor entrance, and after flow field optimization, the uniformity of ammonia distribution becomes the key factor influencing the system’s denitrification performance. Based on the actual structural parameters of a power plant’s SCR denitrification system, a three-dimensional model was established using SCDM software. After meshing in Fluent Meshing, a program was developed in MATLAB with ammonia injection velocity and concentration as variables, and Ansys Fluent was employed for calculations. This approach was used to investigate the ammonia distribution patterns at the SCR reactor entrance under various partitioning schemes, focusing on the injection characteristics in each partition.

## Results

### Geometric structure

**Figure 1:**
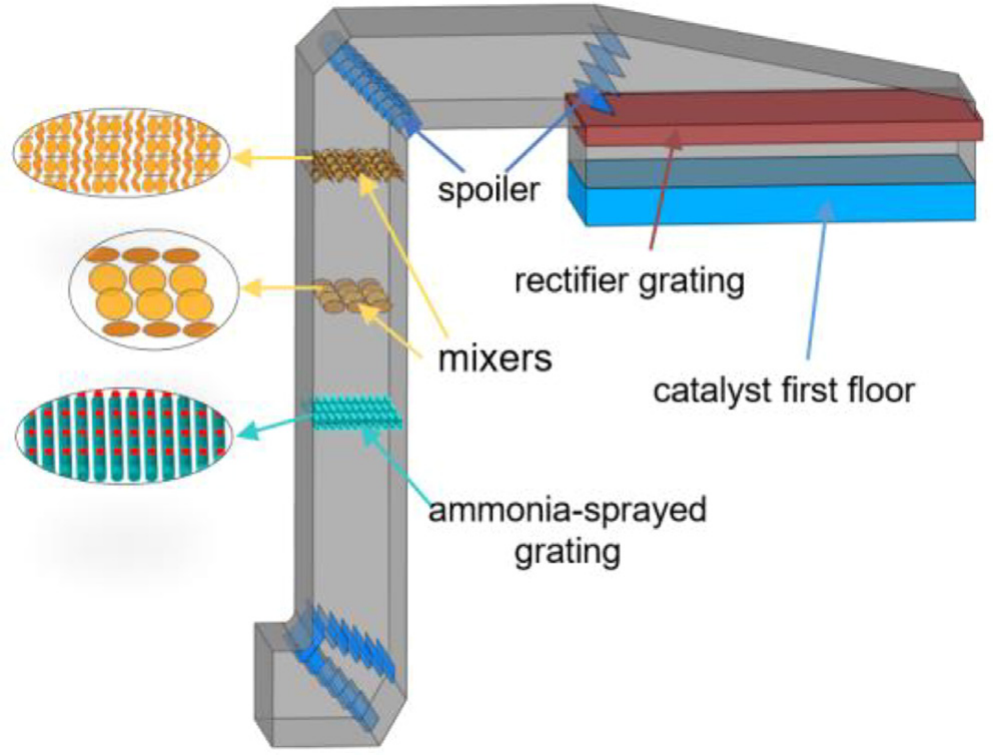
The figure of SCR denitrification system geometric model

Using the SCR denitrification system of a 660 MW unit at a power plant as the research object, the modeled structure extends from the entrance of the SCR denitrification system to the entrance of the SCR reactor. The flue gas enters the system through a horizontal inlet. At the first turn, two sets of guide vanes, each consisting of seven blades, are positioned. The first set of guide vanes has a horizontal section of 200 mm, extending at a 45° angle for 580 mm, followed by the second set, inclined at an angle of 44.9° to the vertical plane, with a length of 200 mm and a vertical extension of 500 mm. Guided by the vanes, the flue gas flows upward. As the flue gas ascends, ammonia is injected by the ammonia injection grid and preliminarily mixed with the gas. The nozzles within the grid have a diameter of 160 mm. To enhance mixing further, a set of 3 × 4 circular mixers, each with a diameter of 850 mm, is installed 4,650 mm above the ammonia injection grid. Further upward, a set of 8 × 16 circular mixers, each with a diameter of 420 mm, is positioned 5,260 mm above the first set. Both sets of mixers are installed at a 45° angle to ensure thorough mixing of flue gas and ammonia. Continuing to the second turn, a set of nine curved guide vanes is installed, followed by a set of four guide vanes at the third turn, each 1,000 mm in length and inclined at an angle of 21.5° to the horizontal plane. Finally, after passing through the straightening grid, the mixed flue gas adjusts its flow direction and velocity distribution to ensure uniform entry into the catalyst layer for denitrification reactions. A three-dimensional 1:1 scale model was constructed in SCDM, and the corresponding geometric model is presented in Figure 1. The boundary parameters in the model and the composition of the working fluid are shown in Tables 1 and 2.

**Table 1:** The inlet boundary conditions of SCR denitrification system

Name	Flue gas flow (t/h)	Flue gas density (kg/Nm^3^)	Cross-sectional area (m^2^)	Entrance velocity (m/s)
Value	2941.26	1.333	54.03	11.344

**Table 2:** The flue gas components at SCR inlet

Components	CO_2_	O_2_	N_2_	H_2_O	NO	SO_2_
Value	14.08	3.96	74.69	6.85	0.054	0.3641

### Validation of the model

To verify the accuracy of the model, the simulation data were compared with actual operating parameters at the inlet of the SCR reactor. As shown in Table 3, the temperature, NO content, and O_2_ content simulated by the model at the inlet of the SCR reactor show minimal deviation from the actual parameters, indicating the model’s consistency with the real SCR denitrification process.

**Table 3:** Comparison of simulation results and actual parameters

Name	Unit	Simulated value	Actual value
Temperature	K	630.15	675.28
O_2_ content	%	3.92	3.85
NO content	mg/m^3^	658	623.14

### Divisional ammonia injection scheme

**Figure 2:**
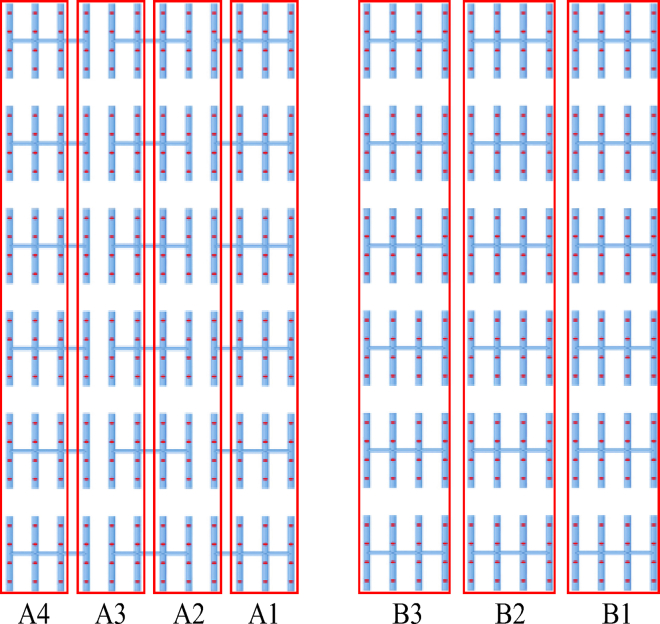
Two schemes of subarea ammonia injection under linear subarea Divide the entire ammonia injection area linearly into four regions: A1, A2, A3, and A4, as scheme I. Divide the entire ammonia injection area linearly into three regions: B1, B2, and B3, as scheme II.

The area-controlled ammonia injection is also called precise ammonia injection. Its essence is to divide the original ammonia injection area in SCR denitrification system into different zones, and each zone is controlled by a single ammonia injection branch pipe. This method is beneficial to improve the uniformity of ammonia injection. In order to reduce the ammonia escape in SCR denitrification system, the original ammonia injection mode was changed into the district control ammonia injection grid mode. Achieving a homogeneous mixing of ammonia and flue gas at the same time requires a study of the flow pattern of ammonia. Based on the actual needs of the power plant, the flow characteristics of ammonia in the SCR denitrification system are studied for each partition under different partitioning methods, and the adopted partitioned ammonia spraying scheme is shown in Figure 2.

## Discussion

### The effect of ammonia spraying velocity and concentration in partition scheme I on the distribution of ammonia concentration

**Figure 3:**
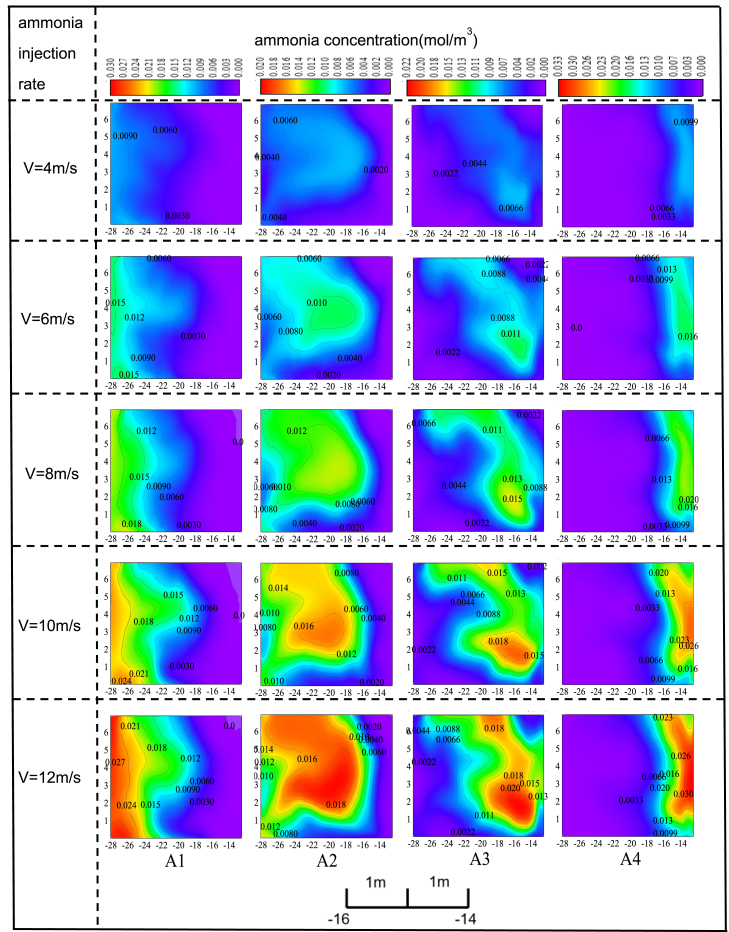
The change of ammonia concentration distribution at the inlet of SCR reactor with ammonia injection speed under the scheme I Under scheme I, the impact of individual ammonia injection in regions A1, A2, A3, and A4 on the ammonia concentration distribution at the catalyst inlet was studied when the ammonia injection concentration was maintained at 1% and the injection velocity was increased from 4 m/s to 12 m/s. In the figure, each coordinate interval corresponds to 1 m of the study object.

In scheme I, with the ammonia concentration from the ammonia grid maintained at 1%, the distribution of ammonia concentration at the inlet of the SCR reactor varies depending on the ammonia spraying velocity, as shown in Figure 3. Figure 3 shows that the ammonia spraying characteristics in area A1 primarily influence the left side of the SCR reactor inlet. As the ammonia spraying velocity increases from 4 m/s to 12 m/s, no significant change is observed in the ammonia concentration distribution at the SCR reactor inlet. In contrast, the ammonia concentration in the corresponding area gradually increases as the velocity increases. This may be due to the increased ammonia spraying velocity causing the ammonia to reach the SCR reactor inlet more quickly, thus reducing the mixing and diffusion time with the flue gas during the flow process. As a result, the ammonia concentration in a specific area at the reactor inlet increases as the ammonia spraying velocity rises.

The ammonia spraying characteristics in area A2 primarily influence the central region at the SCR reactor inlet. An increase in ammonia spraying velocity from 4 m/s to 12 m/s leads to a gradual rise in ammonia concentration in the central region, accompanied by a phenomenon of localized ammonia concentration distribution. Therefore, when designing the ammonia spraying system, it is advisable not to set the spraying velocity too high, as this may increase variability in ammonia concentration distribution at the SCR reactor inlet and result in a higher ammonia slip rate.

The ammonia spraying characteristics in area A3 primarily influence the central-right region of the SCR reactor inlet. As the ammonia spraying velocity increases from 4 m/s to 12 m/s, the maximum ammonia concentration in the corresponding region at the SCR reactor inlet rises from 0.0066 mol/m³ to 0.020 mol/m³. Ammonia is primarily concentrated in the lower-right region of the SCR reactor inlet, and increasing the ammonia spraying velocity does not significantly affect the ammonia distribution area at the reactor inlet.

The ammonia spraying characteristics in area A4 primarily influence the far-right region of the SCR reactor inlet. As the ammonia spraying velocity in area A4 increases from 4 m/s to 12 m/s, the maximum ammonia concentration in the corresponding region at the SCR reactor inlet rises from 0.0099 mol/m³ to 0.030 mol/m³, indicating a significant increase in concentration. However, due to the influence of the wall surface, the ammonia concentration distribution area does not change significantly and remains on the right side of the SCR reactor inlet.

**Figure 4:**
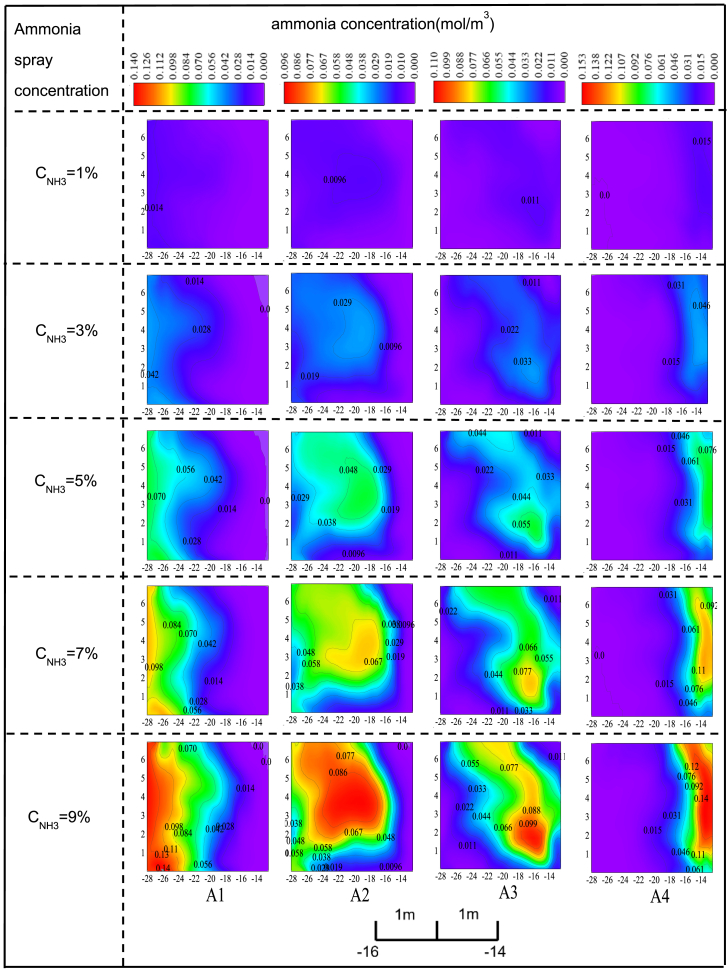
The change of ammonia concentration distribution at the inlet of SCR reactor with injection ammonia concentration under the scheme I Under scheme I, the impact of individual ammonia injection in regions A1, A2, A3, and A4 on the ammonia concentration distribution at the catalyst inlet was studied when the ammonia injection velocity was maintained at 6 m/s and the injection concentration was increased from 1% to 9%. In the figure, each coordinate interval corresponds to 1 m of the study object.

In scheme I, maintaining an ammonia spray velocity of 6 m/s, the distribution of ammonia concentration at the entrance of the SCR reactor varies with the ammonia concentration, as illustrated in Figure 4. Figure 4 shows that the ammonia concentration in area A1 increases from 1% to 9%, and the maximum ammonia concentration in the corresponding region at the SCR reactor entrance rises from 0.014 mol/m³ to 0.140 mol/m³. The area covered by the ammonia distribution expands from one-third to one-half of the original region. The increase in ammonia concentration occurs because other factors remain constant, and raising the ammonia concentration in area A1 naturally causes the concentration at the reactor entrance to rise. The expansion of the ammonia distribution area results from the increased concentration, which creates a larger concentration gradient, leading to the rapid diffusion of ammonia. As a result, the area covered by ammonia at the reactor entrance becomes broader.

The ammonia concentration in area A2 increases from 1% to 9%. The increased concentration gradient accelerates ammonia diffusion, leading to a significant expansion of the ammonia distribution area. Additionally, within the affected region at the SCR reactor entrance, the ammonia concentration distribution is noticeably asymmetrical from left to right. The ammonia distribution is more uniform on the left side, whereas the right side exhibits a more pronounced variation in concentration.

The increase in ammonia concentration in area A3 does not lead to significant changes in the ammonia distribution area at the SCR reactor entrance. This may be attributed to severe turbulence near the center of the flue, where the gas flow is highly complex. Therefore, even though there is a larger ammonia concentration gradient in this region, the ammonia distribution area does not undergo substantial change. Additionally, a concentrated distribution of ammonia is observed at the SCR reactor entrance. Increasing the ammonia spray concentration in area A3 from 1% to 9% raises the maximum ammonia concentration in the corresponding region from 0.011 mol/m³ to 0.099 mol/m³. However, further analysis reveals that increasing the ammonia concentration not only fails to expand the ammonia distribution area but also exacerbates the issue of excessively concentrated ammonia in specific regions at the reactor entrance, which can affect the denitrification performance of the SCR system. Therefore, when designing precise ammonia spraying for actual SCR denitrification systems in power plants, this factor should be carefully considered.

When the ammonia concentration in area A4 increases from 1% to 9%, a significant rise in ammonia concentration is observed at the SCR reactor entrance. Due to the influence of the wall surface, the corresponding ammonia concentration distribution areas are confined to the right side of the SCR reactor entrance. Increasing the ammonia spray concentration in area A4 from 1% to 9% will result in a significant rise in the maximum ammonia concentration near the SCR reactor entrance, from 0.015 mol/m³ to 0.140 mol/m³. Furthermore, the ammonia concentration distribution area will gradually expand as the ammonia spray concentration increases. When the ammonia spray concentration in area A4 is 1%, the main ammonia distribution area is located near the right side of the SCR reactor entrance, accounting for approximately one-fifth of the entrance area. However, when the ammonia spray concentration in area A4 increases to 9%, the ammonia concentration distribution area accounts for about one-third of the reactor entrance.

### The effect of ammonia spraying velocity and concentration in partition scheme II on the distribution of ammonia concentration

**Figure 5:**
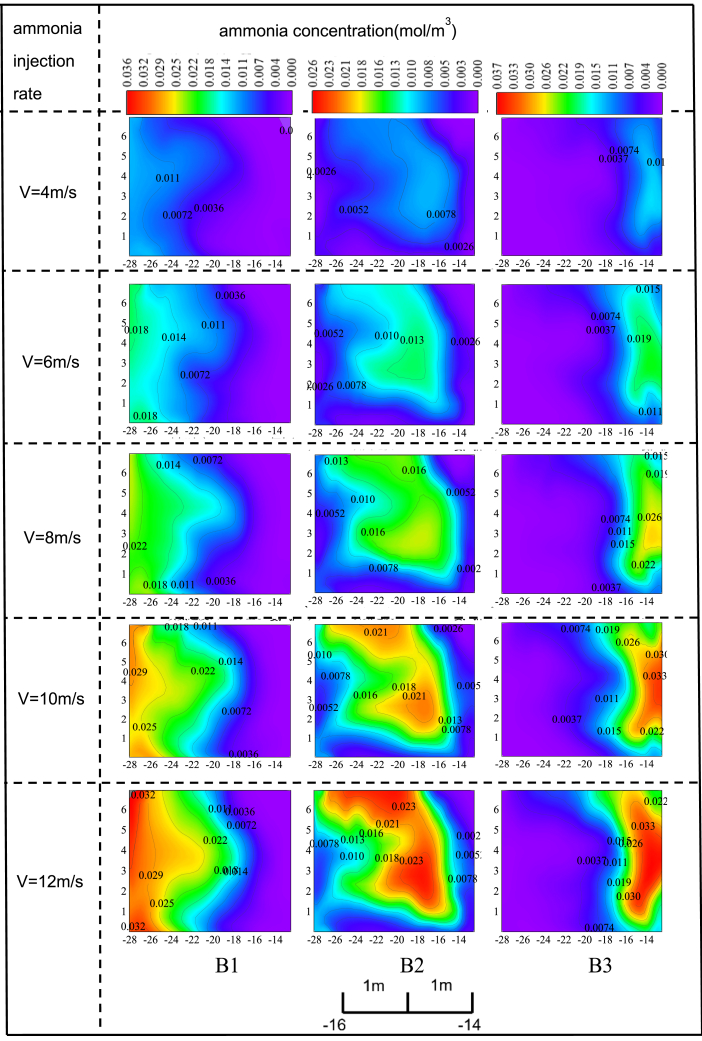
The change of ammonia concentration distribution at the inlet of SCR reactor with ammonia injection speed under the scheme II Under scheme II, the impact of individual ammonia injection in regions B1, B2, and B3 on the ammonia concentration distribution at the catalyst inlet was studied when the ammonia injection concentration was maintained at 1% and the injection velocity was increased from 4 m/s to 12 m/s. In the figure, each coordinate interval corresponds to 1 m of the study object.

In scheme II, with the ammonia concentration sprayed by the ammonia grid maintained at 1%, the distribution of ammonia concentration at the SCR reactor entrance varies with changes in ammonia spray velocity, as shown in Figure 5. In scheme II, the ammonia spraying characteristics in area B1 primarily influence the left half of the SCR reactor entrance. Compared to scheme I, the distribution range of ammonia concentration in area B1 is broader. Therefore, although using a more extensive zoning scheme for the ammonia grid simplifies operation and reduces modification costs, it decreases the ability to precisely control the ammonia concentration distribution in specific regions at the SCR reactor entrance. As the ammonia spray velocity in area B1 increases from 4 m/s to 12 m/s, the ammonia distribution area at the SCR reactor entrance correspondingly expands. When the spray velocity reaches 12 m/s, the ammonia concentration distribution area occupies approximately half of the SCR reactor entrance. Furthermore, increasing the spray velocity does not result in an overly concentrated ammonia distribution in the corresponding area of the SCR reactor entrance; the overall distribution remains relatively uniform.

The ammonia spraying characteristics in area B2 primarily influence the central region at the SCR reactor entrance. Increasing the ammonia spray velocity in area B2 not only increases the ammonia concentration but also significantly expands the ammonia distribution area. When the ammonia spray velocity in area B2 increases to 12 m/s, the ammonia distribution area expands to approximately 1.5 times its original size. This is because, with other factors remaining constant, the influence of a larger zoning scheme on the distribution of ammonia concentration at the SCR reactor entrance outweighs the effect of complex turbulence in the central region. Therefore, the ammonia distribution area gradually increases as the ammonia spray velocity in area B2 rises. Furthermore, since the ammonia distribution area is broad at this stage, there is no issue with the ammonia concentration becoming overly concentrated.

The ammonia spraying characteristics in area B3 primarily influence the right side of the SCR reactor entrance. When the ammonia spray velocity in area B3 increases from 4 m/s to 12 m/s, a significant concentration of ammonia develops near the wall. At a spray velocity of 4 m/s, the maximum ammonia concentration is 0.011 mol/m³. When the spray velocity increases to 12 m/s, the maximum ammonia concentration reaches 0.026 mol/m³, with the area of maximum concentration almost entirely concentrated near the center of the right wall. This indicates that increasing the spray velocity in area B3 can lead to excessive ammonia concentration near the wall, potentially affecting the efficiency and performance of the SCR system. Increasing the ammonia spray velocity in area B3 also leads to an expansion of the ammonia concentration distribution area, though this expansion primarily occurs near the wall surface. This may be due to the complex turbulence in the central region and the fact that area B3 is not the primary factor influencing the ammonia concentration distribution in that region.

**Figure 6:**
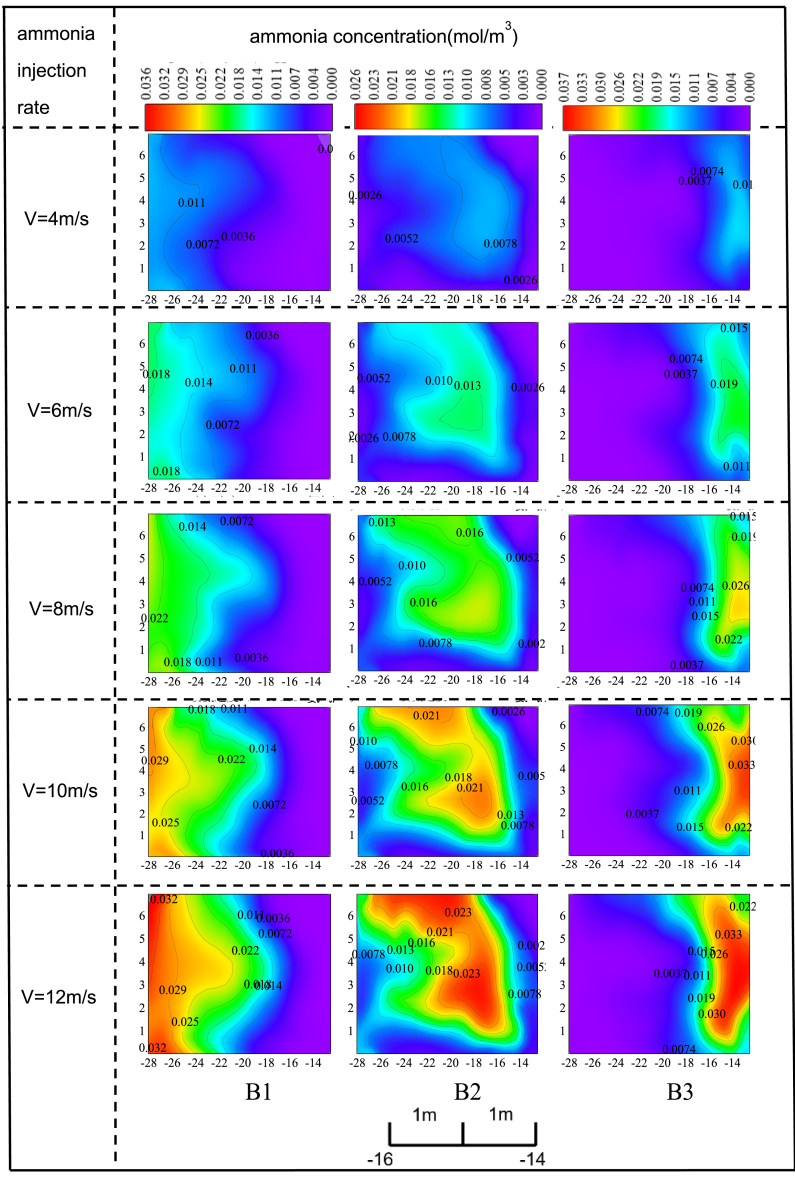
The change of ammonia concentration distribution at the inlet of SCR reactor with injection ammonia concentration under the scheme II Under scheme II, the impact of individual ammonia injection in regions B1, B2, and B3 on the ammonia concentration distribution at the catalyst inlet was studied when the ammonia injection velocity was maintained at 6 m/s and the injection concentration was increased from 1% to 9%. In the figure, each coordinate interval corresponds to 1 m of the study object.

In scheme II, with the ammonia spray velocity maintained at 6 m/s, the distribution of ammonia concentration at the SCR reactor entrance varies with changes in ammonia concentration, as shown in Figure 6. It is observed that when the ammonia spray concentration in area B1 increases from 1% to 9%, there is no significant change in the ammonia distribution area. At an ammonia spray concentration of 1%, the ammonia distribution area accounts for about half of the SCR reactor entrance. When the ammonia spray concentration increases to 9%, the ammonia distribution area remains approximately half of the SCR reactor entrance. Although the ammonia concentration gradient increases with the rise in ammonia spray concentration, the overall ammonia distribution remains relatively uniform, with no issues of localized concentration. This is likely due to the large ammonia distribution area, where ammonia diffuses evenly and concentration changes are gradual. As a result, no localized concentration of ammonia occurs.

When the ammonia spray concentration in area B2 increases from 1% to 9%, there is a gradual accumulation of ammonia at the SCR reactor entrance, though this issue is not significant. At an ammonia spray concentration of 1% in area B2, the maximum ammonia concentration at the center of the SCR reactor entrance is approximately 0.013 mol/m³. When the ammonia spray concentration in area B2 increases to 9%, the maximum ammonia concentration at the center of the SCR reactor entrance reaches 0.120 mol/m³. Additionally, the ammonia distribution area gradually expands outward as the ammonia spray concentration in area B2 increases, occupying a larger region. When the ammonia spray concentration in area B2 reaches 9%, the ammonia distribution area occupies three-quarters of the SCR reactor entrance.

**Figure 7:**
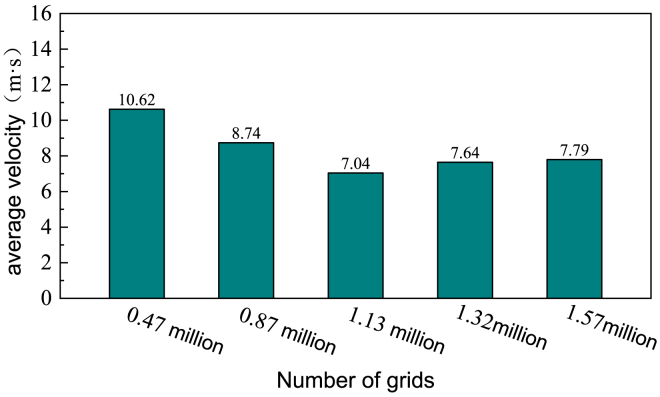
The average inlet velocity before the first layer of catalyst under different grid numbers

The increase in ammonia spray concentration in area B3 leads to a phenomenon in the ammonia concentration distribution at the SCR reactor entrance similar to that caused by the aforementioned spray velocity factor. As the ammonia spray concentration increases, the ammonia distribution area also expands, and no significant issue with local overconcentration is observed.The conclusion was drawn after grid independence verification, as shown in Figure 7. It has practical significance for reducing pollutant emissions in coal-fired power plants.

### Conclusion

Through simulating the effects of various zoned ammonia injection schemes on the flow field distribution at the entrance of the selective catalytic reduction (SCR) reactor, we uncovered the interaction mechanisms between flue gas flow characteristics and ammonia distribution. The main conclusions of this study are as follows:

In scheme I, the ammonia injection characteristics of each zone significantly impact different areas of the SCR reactor inlet. The ammonia injection characteristics of Zone A1 primarily influence the left side of the catalyst inlet, whereas zones A2, A3, and A4 respectively affect the central area, the area slightly right of the center, and the far-right region. An increase in ammonia injection velocity results in excessive ammonia concentration in specific areas, particularly in zones A1 and A2. Additionally, an increase in ammonia concentration expands the ammonia distribution area, especially in zone A2. However, in zone A3, increased ammonia injection velocity does not significantly impact the distribution area, though a rise in ammonia concentration causes a more pronounced concentration issue. In zone A4, an increase in ammonia injection velocity noticeably raises ammonia concentration, but due to wall influence, there is no significant change in the distribution area.

In scheme II, the region influenced by the ammonia injection characteristics at the SCR reactor inlet is broader compared to scheme I. Although a wider ammonia injection grid division may reduce the ability to precisely control ammonia concentration in specific areas, it helps prevent overly concentrated ammonia distribution in localized regions. An increased ammonia injection rate in zone B1 enlarges the ammonia distribution area, though the concentration remains relatively uniform. An increased ammonia injection rate in zone B2 expands the ammonia concentration distribution area, and a rise in ammonia concentration causes the distribution to gradually spread outward. In zone B3, an increase in both ammonia injection rate and concentration expands the distribution area, potentially causing overly concentrated ammonia in localized areas.

These findings provide scientific evidence for the precise design of ammonia injection in power plants, optimizing denitrification system performance, ensuring compliance with environmental standards, and improving the system’s economy and reliability. In designing the ammonia injection scheme, only linear zoning was considered, whereas other schemes were not explored in detail. Further division of zoning schemes could lead to improved ammonia control in power plants, achieving better results.

### Limitations of the study

Although this study provides new insights and data on precision ammonia injection in coal-fired power plants, certain limitations remain. In designing the ammonia injection scheme, only a linear partitioning scheme was employed, with additional schemes planned for future investigation.

## Resource availability

### Lead contact

Further information and requests for resources should be directed to and will be fulfilled by the lead contact, Dehong Gong (dhgong@gzu.edu.cn).

### Materials availability

This study did not generate new unique materials.

### Data and code availability

• All data reported in this paper will be shared by the lead contact upon request.
• This paper does not report the original code.
• Any additional information required to reanalyze the data reported in this paper is available from the lead contact upon request.

## Acknowledgments

This work was supported by the Science and Technology Program of Guizhou Jinyuan Co. (138006JX0120220018); Guizhou Provincial Science and Technology Program Project (Qiankehe Support [2022] General 018); and Scientific Research Program for Introducing Talents of Guizhou University (Gui Da Ji He Zi [2022] No. 72).

## Author contributions

J.D.Z., investigation, formal analysis, and writing—original draft. Z.Q.Y., conceived the idea of visualization. D.H.G., project administration, resources, supervision, and validation. W.Q., review & editing. Q.L.L., analyzed data. All authors discussed the results and approved the article.

## Declaration of interests

The authors declare no competing interests.

## STAR★Methods

### Key resources table

**Table:** 

REAGENT or RESOURCE	SOURCE	IDENTIFIER
**Deposited data**		

Numerical Simulation Dataset	This paper	N/A

**Software and algorithms**		

Ansys fluent 13.0	ANSYS	https://www.ansys.com/
SpaceClaim2010	ANSYS	https://www.ansys.com/
MATLAB 6.0	MathWorks	http://b.zhr33.cn/matlab/

### Method details

#### Model assumptions

(1) Due to the small size of each pipe rack support, beams and other components inside the flue, the influence on the flow field inside the flue is not significant, so it will be ignored, and the influence of the thickness of the deflector plate, mixer and other components inside the flue on the flow field will not be considered;
(2) The effect of fly ash on the flow field is not considered because the flue gas has been de-ash due to the relevant modifications carried out in the power plant before entering into the SCR denitrification system;
(3) The flue gas is treated as an incompressible ideal gas, and the effects of chemical reactions occurring during the flow of the various components of the flue gas are not taken into account;
(4) Because of the low temperature of the flue gas, the effect of radiative heat transfer is not considered; since the actual system is better insulated, the whole system is assumed to be in an adiabatic state, and air leakage from the device is not considered.

#### Mathematical modeling

The flow of flue gases is governed by three basic equations, namely, conservation of mass, conservation of momentum and conservation of energy.

The mass conservation equation^39^:

(Equation 1) $$\frac{\partial \rho }{\partial t}+\nabla \left(\rho \vec{v}\right)=S_{m}$$

Where: ρ is the density of the fluid, kg/m^3^; t is the time, s; $\vec{v}$ is the velocity of the fluid, m/s; $S_{m}$ is the mass source term, kg.

The conservation of momentum equation^40^:

(Equation 2) $$\frac{\partial }{\partial t}\left(\rho \vec{v}\right)+\nabla \left(\rho \vec{v}\vec{v}\right)=-\nabla p+\left[\mu \left(\nabla \vec{v}+{\left(\nabla \vec{v}\right)}^{T}\right)-\frac{2}{3}\nabla \vec{v}I\right]+\rho \vec{g}+\vec{F}$$

Where: p is the static pressure, Pa; μ is the dynamic viscosity, (N·s)/m^2^; $\rho \vec{g}$ is the force of gravity acting on a unit of micrometric fluid, N; I is the unit tensor; $\vec{F}$ is the external force acting on a unit of fluid, N.

The equation of conservation of energy:

(Equation 3) $$\nabla \left[\rho \vec{v}\left(\int_{T_{ref}}^{T}c_{P}dT\right)\right]=\nabla \left[\left(k+k_{t}\right)\nabla T-\sum_{j}h_{j}{\vec{J}}_{j}\right]+S_{h}$$

Where: k is the laminar thermal conductivity, W/(m·K); $k_{t}$ is the turbulent heat conductivity,W/(m·K); $\sum_{j}h_{j}{\vec{J}}_{j}$ is the sensible enthalpy transfer due to diffusion of components, J/kg; $S_{h}$ is a volumetric heat source term, J.

Its application in engineering practice often requires simplification of the equations by adopting a number of assumptions. Among them, the standard k - ε two-equation turbulence model generated by adopting Boussinesq’s assumption is widely used in engineering because of its simple form, easy to solve, and relatively high accuracy in predicting the main flow and pressure of non-separating shear turbulence. Its mathematical form expressed as a tensor is shown below.

In the standard k - ε double equation, the turbulent kinetic energy k and the turbulent dissipation rate ε are defined as:

(Equation 4) $$k=0.5\bar{u_{i}^{2}}$$

(Equation 5) $$\varepsilon =\frac{\mu }{\rho }\bar{{\left(\frac{\partial u_{i}}{\partial x_{j}}\right)}^{2}}$$

The transport equation for the turbulent kinetic energy k and the turbulent dissipation rate ε is expressed as follows^41^:

(Equation 6) $$\frac{\partial }{\partial t}\left(\rho k\right)+\frac{\partial }{\partial x_{i}}\left(\rho ku_{i}\right)=\frac{\partial }{\partial x_{j}}\left[\left(\mu +\frac{\mu_{t}}{\sigma_{k}}\right)\frac{\partial k}{\partial x_{j}}\right]+G_{k}+G_{b}-\rho \varepsilon -Y_{M}+S_{k}$$

(Equation 7) $$\frac{\partial }{\partial t}\left(\rho \varepsilon \right)+\frac{\partial }{\partial x_{i}}\left(\rho \varepsilon u_{i}\right)=\frac{\partial }{\partial x_{j}}\left[\left(\mu +\frac{\mu_{t}}{\sigma_{\varepsilon }}\right)\frac{\partial \varepsilon }{\partial x_{j}}\right]+C_{1\varepsilon }\frac{\varepsilon }{k}\left(G_{k}+C_{3\varepsilon }G_{b}\right)-C_{2\varepsilon }\rho \frac{\varepsilon^{2}}{k}+S_{\varepsilon }$$

Where: $\mu_{t}$ is the turbulent viscosity, $\mu_{t}=\rho C_{\mu }\frac{k^{2}}{\varepsilon }$; $G_{k}$ is the production term for the turbulent kinetic energy due to the mean velocity gradient, $G_{k}=-\rho \bar{u_{i}^{\prime }u_{j}^{\prime }}\frac{\partial u_{j}}{\partial x_{i}}$; $G_{b}$ is the buoyancy-induced turbulent energy production term, $G_{b}=\beta g_{i}\frac{\mu_{t}}{Pr_{t}\frac{\partial T}{\partial x_{i}}}$, $\beta =-\frac{1}{\rho }{\left(\frac{\partial \rho }{\partial T}\right)}_{p}$; $Y_{M}$ is the contribution of pulsation expansion in compressible turbulence, $Y_{M}=2\rho \varepsilon M_{t}^{2}$; $C_{1\varepsilon }$、 $C_{2\varepsilon }$、 $C_{\mu }$、 $C_{3\varepsilon }$ is the empirical constant, $C_{1\varepsilon }=1.44$、 $C_{2\varepsilon }=1.92$、 $C_{\mu }=0.09$、 $C_{3\varepsilon }=0.09$; $\sigma_{k}$ is the Prandtl number corresponding to k, $\sigma_{k}=1.0$; $\sigma_{\varepsilon }$ is the Prandtl number corresponding to ε, $\sigma_{\varepsilon }=1.3$.

Since flue gases often contain oxygen, ammonia, nitrogen and NOx components, and there is mutual diffusion between the components, the physical process can be described using a component transport model. Its mathematical form is expressed as follows^16^:

(Equation 8) $$\frac{\partial }{\partial t}\left(\rho Y_{i}\right)+\nabla \cdot \left(\rho \vec{v}Y_{i}\right)=-\nabla \cdot \vec{J_{i}}+R_{i}+S_{i}$$

Where: $Y_{i}$ is the mass fraction of component i; $S_{i}$ is the additional generation rate of component i due to the source term, mol/(m^3^·s); $R_{i}$ is the net rate of production of component i due to chemical reactions, mol/(m^3^·s). $\vec{J_{i}}$ is the diffusive flux of component i induced by a temperature gradient and a concentration gradient. When the fluid is in turbulent flow, it is often calculated by the following equation:

(Equation 9) $$\vec{J_{i}}=-\left(\rho D_{i,m}+\frac{\mu_{t}}{Sc_{t}}\right)\nabla Y_{i}-D_{T,i}\frac{\nabla T}{T}$$

Where: $D_{i,m}$ is the mass diffusion coefficient of component i in the mixture, m^2^/s; $D_{T,i}$ is the temperature diffusion coefficient of component i in the mixture, m^2^/s; $Sc_{t}$ is the turbulent Schmidt number, $Sc_{t}=\mu_{t}/\rho D_{t}$.

#### Meshing

After establishing the geometric model, it was imported into Fluent Meshing software for grid generation. Due to the complex structures of components such as the ammonia injection grid, guide vanes, mixers, and straightening grids, the mesh density in these areas was refined. Given the critical role of boundary layer grids in flow field simulation, the mesh density in the boundary layer was also refined. The polyhedral meshing method was applied to the entire model. Based on this approach, five grid sets containing 0.46 million, 0.87 million, 1.13 million, 1.32 million, and 1.57 million cells were generated for grid independence validation. The calculated average velocity at the SCR reactor inlet for each grid set is shown in Figure 7. It was observed that when the grid count reached 1.57 million, the average inlet velocity stabilized. Therefore, the grid count was set to 1.57 million.

#### Boundary conditions

Based on the actual operating conditions of the flue gas in the SCR denitrification system, the boundaries involved in the flue gas flow—including the flue gas inlet, ammonia injection grid inlet, flue gas outlet, ammonia injection grid pipe wall, flue wall, flow guiding plate wall, and mixer wall—were defined accordingly. A velocity inlet boundary condition was applied at the flue gas inlet, with the inlet velocity set at 11.344 m/s. To ensure simulation accuracy, the parameters of the flue gas, including its constituents and concentrations, were specified in detail. The specific values are presented in Tables 1 and 2.

The velocity inlet boundary condition was also applied at the ammonia grid inlet, with the ammonia volume concentration set to 5% and the flow medium defined as a mixture of air and ammonia. The inlet velocity of the ammonia grid was set at 8.94 m/s, calculated based on the actual ammonia injection volume and the orifice diameter. The flue gas outlet was defined as a free-flow boundary condition, where no predefined values were set, and the distribution of physical quantities at this boundary was obtained through iterative software calculations. The walls of the flue, ammonia injection grid, and inflow device were assigned No-Slip-Wall boundary conditions.

#### Solution parameters

Due to the symmetric structure of the system along the depth of the flue, symmetry boundary conditions are applied at the corresponding locations. The pressure-velocity coupling method uses the SIMPLE (Semi-Implicit Method for Pressure-Linked Equations) algorithm during the simulation process. The gradient term discretization uses the Least Squares Cell-Based method, the pressure term discretization uses the Second Order method, and the convection terms for turbulent kinetic energy and dissipation rate use the First Order method. All other variables are discretized using the Second Order method. The residuals for each equation are set to 1.0 × 10^−6^. Additionally, monitoring variables are set for average pressure and velocity at the SCR outlet. Once the monitoring variables stabilize, the internal flow field is considered converged, allowing for data extraction and analysis.

### Quantification and statistical analysis

This study utilizes custom MATLAB code (see Data S1) to interface with Fluent for executing simulation calculations. Following the completion of calculations, model outlet data is imported into MATLAB to generate visualizations, which are subsequently manually compiled into final analysis charts.
