# Supplementary material for: Ammonia distribution characteristics at the selective catalytic reduction reactor inlet with linear partitioning
Source: iScience. 2024 Dec 12;28(1):111588. doi: 10.1016/j.isci.2024.111588 (PMC11732506; doi:10.1016/j.isci.2024.111588)
Supplement: Document S1. Data S1 [file mmc1.pdf]

iScience, Volume *28*

Supplemental information

Ammonia distribution characteristics at the selective catalytic reduction reactor inlet with linear partitioning

Jiangdong Zhu, Zongquan Ye, Dehong Gong, Qian Wang, and Qingling Luo

```
clc;
clear;
% Set up parallel computing%
CoreNum=10; % Set the number of CPU cores on the machine%
if isempty(gcp('nocreate'))
      parpool(CoreNum);
end
T(1,:)=(0.8:0.1:1.2);
u=length(T);
parfor x=1:u
   a=(0:0.1:0.5)*T(1,x);
   n=length(a);
   h=zeros(1,n);
   m=12960;
for j=1:n
   b=zeros(m,4);
   b(:,3)=normrnd(T(1,x),a(j),1,m)';
   b(:,2)=0.00446;
   b(:,4)=653.15;
   b(:,1)=2;
c=zeros(m,1);
d=zeros(m,1);
o=0;
k=0;
      for i=1:m
         [c(i),d(i)]=shuju(b(i,1),b(i,2),b(i,3),b(i,4));
         if c(i)<0 || c(i)>1 || isnan(c(i))
            c(i)=0;
            o=o+1;
         end
         if d(i)<0 || d(i)>1 || isnan(d(i))
            d(i)=0;
            k=k+1;
         end
         i
      end
   a(j)=roundn(100*sum(c)/(m-o),-2);
   h(j)=roundn(100*sum(d)/(m-k),-9);
end
H(x,:)=a(1,:)
K(x,:)=h(1,:)
end
figure(1)
plot(H)
```

```
figure(2)
plot(K)
```
